# Strengthening the Collection and Use of Disaggregated Data to Understand and Monitor the Risk and Burden of COVID-19 Among Racialized Populations

**DOI:** 10.1007/s42650-021-00050-2

**Published:** 2021-10-04

**Authors:** Josephine Etowa, Ilene Hyman, Charles Dabone, Ikenna Mbagwu, Bishwajit Ghose, Yujiro Sano, Muna Osman, Hindia Mohamoud

**Affiliations:** 1 Faculty of Health Sciences, University of Ottawa, Ottawa, ON Canada; 2 Dalla Lana School of Public Health, University of Toronto, Toronto, ON Canada; 3 Department of Sociology, Nipissing University, North Bay, ON Canada; 4 Ottawa Local Immigrant Partnership, Ottawa, ON Canada

**Keywords:** Population health, COVID-19, Determinants of health, Data collection, Disaggregated data, Immigrants, Racialized populations, Santé des populations, COVID-19, Déterminantes de la santé, Collecte de données, Données désagrégés, Immigrants, Populations racialisées

## Abstract

There is growing evidence that the risk and burden of COVID-19 infections are not equally distributed across population subgroups and that racialized communities are experiencing disproportionately higher morbidity and mortality rates. However, due to the absence of large-scale race-based data, it is impossible to measure the extent to which immigrant and racialized communities are experiencing the pandemic and the impact of measures taken (or not) to mitigate these impacts, especially at a local level. To address this issue, the Ottawa Local Immigration Partnership partnered with the Collaborative Critical Research for Equity and Transformation in Health lab at the University of Ottawa and the Canadians of African Descent Health Organization to implement a project to build local organizational capacities to understand, monitor, and mitigate the impact of the COVID-19 pandemic on immigrant and racialized populations. This research note describes the working framework used for this project, proposed indicators for measuring the determinants of health among immigrant and racialized populations, and the data gaps we encountered. Recommendations are made to policymakers, and community and health stakeholders at all levels on how to collect and use data to address COVID-19 health inequities, including data collection strategies aimed at community engagement in the collection of disaggregated data, improving methods for collecting and analyzing data on immigrants and racialized groups and policies to enable and enhance data disaggregation.

Résumé

Des plus en plus d’études montrent que le risque et le fardeau des infections à la COVID-19 ne sont pas également répartis dans la population et que les communautés racialisées connaissent des taux de morbidité et de mortalité disproportionnellement plus élevés. Cependant, en raison de l’absence de données ventilés selon le statut ethnique, il est impossible de mesurer comment les communautés immigrantes et racialisées vivent la pandémie et quel est l’impact des mesures prises (ou non) pour atténuer ces effets, surtout à un niveau local. Pour résoudre ce problème, le Partenariat local pour l’immigration d’Ottawa (PLIO) s’est associé au Laboratoire de recherche critique collaborative pour l’équité et la transformation en santé (CO-CREATH) de l’Université d’Ottawa et l’Organisation de la santé des Canadiens d’ascendance africaine (CADHO) aux fins de mettre en œuvre un projet visant à renforcer les capacités organisationnelles locales pour comprendre, surveiller et atténuer l’impact de la pandémie de la COVID-19 sur les populations immigrantes et racialisées. Cette note de recherche décrit le cadre de travail utilisé pour ce projet, les indicateurs proposés pour mesurer les déterminants de la santé chez les populations immigrantes et racialisées, et les lacunes que nous avons identifiés dans les données existants. Des recommandations sont faites aux décideurs politiques et aux acteurs communautaires et de la santé à tous les niveaux sur comment collecter et utiliser les données pour remédier aux inégalités en matière de santé liées à la COVID-19. Ces recommandations font référence aux stratégies de collecte de données visant à impliquer les communautés, à l’amélioration des méthodes de collecte et d’analyse des données sur les immigrants et les groupes racialisés, et aux politiques nécessaires pour permettre et améliorer la désagrégation des données selon le statut ethnique.

## Introduction

Globally, it is well-documented that the risk and burden of COVID-19 is disproportionately higher among immigrant and racialized populations (Hooper et al., 2020; Wang & Tang, 2020; Yaya et al., 2020; CDC, 2021; Kuy et al., 2020). In the USA, Strully et al. (2021) observe that counties with higher percentages of foreign-born residents experience higher rates of COVID-19 infections. In Norway, immigrants experience higher rates of infection and hospitalization than non-immigrants, with alarmingly high rates among Somali, Pakistani, and Iraqi immigrants (Indseth et al., 2020). In Sweden, immigrants from low- and middle-income countries experience higher risk of death from COVID-19 than those born in Sweden (Drefahl et al., 2020). The data for racialized groups is equally grim. In England, the Black community experiences the highest rates of COVID-19 diagnosis, and one-quarter of patients requiring intensive care identify as Black or Asian (Razai et al., 2021). Mackey et al. (2021) reported that African-American and Hispanic populations experience higher rates of infection, hospitalization, and mortality than non-Hispanic white populations.

The risk and burden of COVID-19 are not equally distributed in the Canadian population either. Black communities in Canada experience higher rates of COVID-19-related infections and deaths (Choi & Denice, 2020). Existing data shows that the neighborhoods with the highest proportions (25% or more) of population groups identifying as “racialized” (visible minorities) experience a COVID-19 mortality rate twice as high as neighborhoods with the lowest proportions (less than 1%) of racialized populations (Subedi et al., 2020). Approximately 35 percent of Black Canadians had at least one health condition that increased their risk of severe COVID-19 health outcomes (Statistics Canada, 2020).

Despite this evidence, the Province of Ontario was initially reluctant to collect and release demographic and socioeconomic indices on COVID-19 infections and deaths, considering “all groups of people are equally important to us” (Siddiqi et al., 2020). By contrast to the government response, academic and community groups increasingly viewed the COVID-19 pandemic and its outcomes as a structural issue in Canada that was disproportionately affecting equity-seeking communities, such as immigrants, refugees, women, and people who are from low-income background, have disabilities, and/or are housing insecure (Choi et al., 2021, Etowa & Hyman, 2021). By the end of 2020, the Province of Ontario invested in the possibility of sociodemographic data collection for the vaccine roll-out, and the Federal Government announced a national sociodemographic data collection initiative and a pandemic equity model (McKenzie, 2021). It remains challenging to estimate the degree to which immigrant and racialized communities are experiencing the pandemic and the impact of measures taken (or not) to mitigate these impacts, especially at a local level.

To address this issue, in 2020, the Ottawa Local Immigration Partnership (OLIP) initiated its *Strengthening Disaggregated Sociodemographic Data Related to COVID-19* project to build organizational capacities to understand, monitor, and mitigate the impact of the COVID-19 pandemic on immigrant and racialized populations. As part of this project, OLIP partnered with the Collaborative Critical Research for Equity and Transformation in Health (CO-CREATH) team at the University of Ottawa and the Canadians of African Descent Health Organization (CADHO) to develop a conceptual and operational framework to guide data collection and use. In this research note, we present our working framework, propose indicators for measuring the determinants of health (SDOH) among immigrant and racialized populations, and identify data gaps. We conclude with recommended strategies for community engagement in the collection of disaggregated data, methods for collecting and analyzing data about immigrants and racialized groups, and policies that would enable and enhance data disaggregation in Canada. We also propose that our working framework to address COVID-19 health inequities^1^ is useful and transferable to other geographic or institutional settings.

## Description of Working Framework

The social determinants of health (SDOH) framework is useful to understand how structural determinants such as race and racism determine health outcomes (Wilkinson and Marmot, 2003). Ecological frameworks, such as the socio-ecological model developed by Bronfenbrenner (1979), identify a vast array of layered micro-, meso-, exo-, and macro-level factors that are particularly relevant when addressing health inequities with respect to COVID-19 infection and outcomes among racialized populations. *Macro-level determinants* include global contexts, reasons for migration (i.e., voluntary vs. involuntary), immigration policies, resettlement policies, labor market conditions, gender and occupational stereotypes, gender-segregated job markets, rights and entitlements of migrants, housing, economics, and physical and social environments. As a result of immigration policies that exclude migrants with serious medical conditions and select migrants with higher education, language ability, and job skills, characteristics which facilitate social and economic integration, immigrants to Canada are largely a “healthy” group (Kennedy et al., 2006; Biddle et al., 2007; Frisbie et al., 2001). Macro-level determinants are typically operationalized by documenting (1) laws and policies (e.g., income security and distribution, housing, immigration, health); (2) the availability, accessibility, and adequacy of resources and opportunities that influence the distribution of necessary and desired conditions and services to individuals and their ease of uptake; (3) governance and spending (i.e., the amount of spending available and used to support government programs); and (4) social norms and values. Macro-level determinants of health work together to create health inequities because they shape the ways that power, money, and resources are distributed in society, which provide individuals with greater or lesser ability to have control over their health (Commission on Social Determinants of Health, 2008; Blumenshine et al., 2008; PHAC & PPHN, 2018).

*Meso-level determinants* include features and relationships among organizations and institutions at the community level (e.g., geographic, social) that may affect health. They include the spaces and places where people work and interact and may include community/neighborhood characteristics (e.g., community cohesion, social and faith networks and support, social norms, and community values) and access to and quality of local amenities (e.g., public health, recreation, education, transportation, housing). Racism is a prominent form of social exclusion, which is in itself a major determinant of health. There is growing evidence that racism contributes to the declining health among immigrants directly and indirectly through other social determinants (Paradies et al., 2015; De Maio & Kemp, 2010; Hyman, 2009). The work environment is considered by many to be a meso-level determinant of health affecting the health of workers, families, and communities, including those who are precariously employed (Benach & Muntaner, 2011). Community-level determinants may be operationalized using local data sources (e.g., data on access and use of local services) as well as population and/or neighborhood-level data.

*Micro-level determinants* include those identified in Health Canada’s framework (e.g., age, marital status, income, education, occupation, employment, economic dependency, religion, health behaviors, health beliefs, health literacy, psychological resources) as well as those specific to the migration context, such as age at migration, immigration status, length of stay, family separation, cultural retention, and resettlement stress. Family and individual determinants are commonly operationalized using population data.

Any examination of the determinants of COVID-19-related health inequities among immigrants and racialized populations must also consider the complex issues of the *dynamic interplay between different levels of determinants, intersectionality, and a life course perspective*. For example, pre- and post-COVID-19 health inequities can be reduced by altering social policies, strengthening institutional resources and relationships, and supporting the development of community leaders and key community social settings. Since the determinants of health may be distributed differently and have differential impacts on different population subgroups, the need to consider *intersectionality* and to recognize the multiple layers of factors that contribute to health inequities is critical. For example, the healthy immigrant effect (HIE) refers to the observation that immigrants are often in superior health to the host populations (Kennedy et al., 2006); however, it does not apply to refugees, who often arrive in poor health as a result of exposure to infectious disease, trauma, unhealthy living conditions, precarious financial status, and limited access to quality health care services (Lu & Ng, 2019). The *life course perspective* recognizes that exposures from gestation through childhood, youth, and mid-life affect health in adult and later life as well as health across generations (Kuh & Ben-Shlomo, 2004). The relative good health of immigrants upon arrival is not a guarantee of good health in the long term, and within this group, there are differential rates of transitioning to poor health (Ru & Li, 2021; Ng & Zhang, 2020; Sano & Abada, 2019; Subedi & Rosenberg, 2014; Ng et al., 2005; Newbold, 2005). These are also the groups that are at the highest risk of COVID-19.

**Fig. 1:**
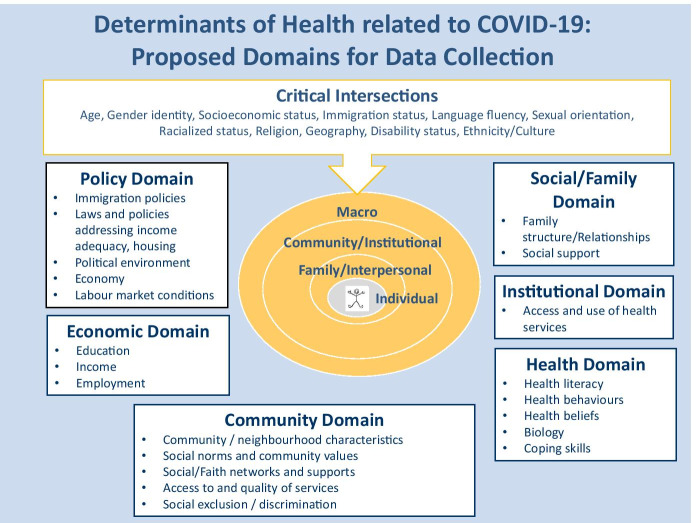
Determinants of health related to COVID-19: a working framework

Figure 1 describes the working framework that guided our work. It includes the multi-level determinants and intersectional factors that influence health outcomes in the current COVID-19 context and the six functional domains that we used to identify our proposed indicators. The policy domain includes indicators related to immigration, poverty reduction, income distribution, economic, housing, employment, and health policies and other macro-level conditions (e.g., labor market). The economic domain includes indicators related to socioeconomic status such as education, employment, occupation, and income. The community domain includes indicators related to the community and work environments people occupy, for example, the social norms of their communities, social engagement, sense of belonging, discrimination, community safety, housing, social exclusion/discrimination, and food insecurity. The institutional domain includes indicators related to access to health, recreational, social programs, services, etc. The social domain includes indicators related to people’s social relationships, e.g., friends and family. The health domain includes indicators related to individual health risk and health-enhancing behaviors.

It is important to further recognize how the evolution and waves of COVID-19 have been structurally and politically constructed and informed. According to the SEM, individuals are nested within families which are nested within multiple communities, which are nested in broader social and political contexts. Since governments have reacted to the COVID-19 pandemic in different ways, individuals have had to make social, economic, and behavioral decisions within these broader political systems. Thus, it is critical to look at the relationship between individual- and structural-level factors and how multi-level social determinants of health influence COVID-19-related outcomes. This type of perspective is critical to inform the type of intersectoral actions necessary to mitigate COVID-19-related risk and burden in immigrant and racialized populations (Logie & Turan, 2020).

### Indicators for Measuring the Determinants of Health Among Immigrant and Racialized Populations

To identify the indicators within the six domains of interest, we relied on previous Canadian work in indicator development. For example, PHAC’s Health Inequalities Tool identified indicators in different domains that can be used to operationalize health inequalities between population groups (Pan-Canadian Health Inequalities Tool, 2017). The Canadian Index for Measuring Integration (CIMI) identified key indicators in four separate domains that may be used to measure inequalities between immigrant and non-immigrant populations in different domains and provides ranks at the provincial and municipal level. The identification of previous scholarly work on indicator selection for a geographic or institutional jurisdiction helps to ensure that the indicators selected have face validity and are reliable and available using existing sources of data.

We developed specific criteria to guide the indicator selection:

- Source reference for the indicator (i.e., which source has recommended/used this indicator)
- An operational definition of the indicator
- The data source available for the indicator
- Whether the data was available at the local (i.e., Ottawa) level
- Whether the data could be disaggregated by immigrant status and racialized status. Note that some indicators could be further disaggregated by length of stay in Canada, first language, and specific racialized identity, depending on type of analyses required and level of geography. Data sets linked to the Longitudinal Immigration Database (IMDB) could be disaggregated by country of origin and immigration class (i.e., refugee, economic, family), but these data were not currently available for public use.

Table 1 presents a summary of the indicators we identified that may be used to measure the correlation of COVID-19 risk and burden among immigrant and racialized populations in Ottawa that may be adapted by other local public and community agencies. It includes indicators for the political domain, such as the existence and implementation of government policies related to immigration, poverty reduction, labor market, and health, that are largely drawn from government documents. Economic domain indicators, such as percent of population without a high school degree and percent of 25- to 64-year-olds in population with a university degree, are largely drawn from Statistics Canada surveys (e.g., Labour Force, Census and General Social Surveys). Community domain indicators, such as percent of population that reports very or somewhat strong sense of belonging to community, are largely drawn from Statistics Canada surveys but include indicators such as crimes against property and people that are drawn from local sources. Some indicators on access to services, such as the percent of the population with a regular medical doctor, are drawn from Statistics Canada surveys, but others, such as the percent of the population with a Community Health Centre within a 50-m walk, are drawn from local sources. Indicators for the health and social domains are largely drawn from Statistics Canada survey data (e.g., Canadian Community Health Survey, General Social Survey).

**Table 1.**
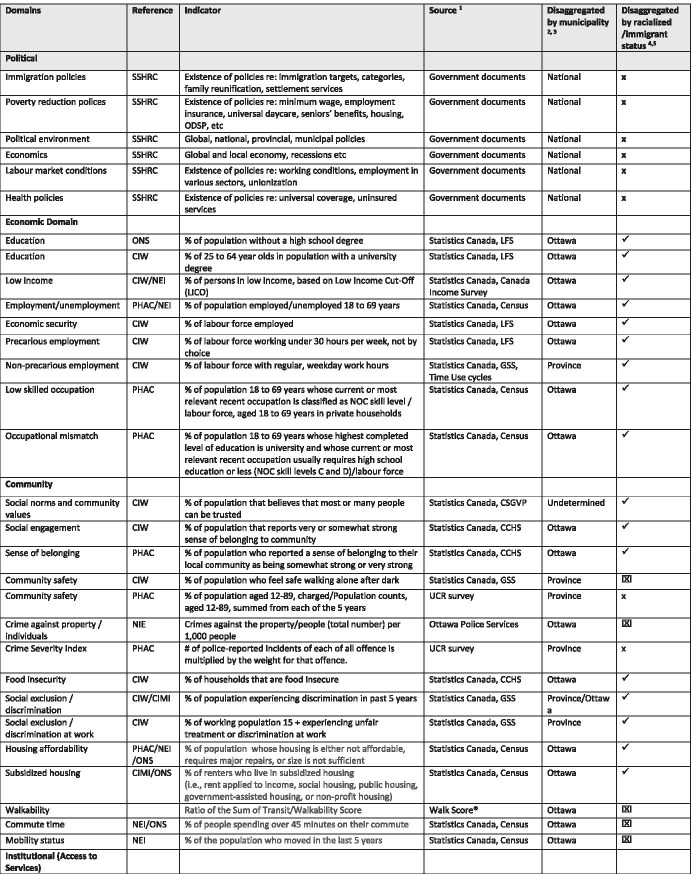

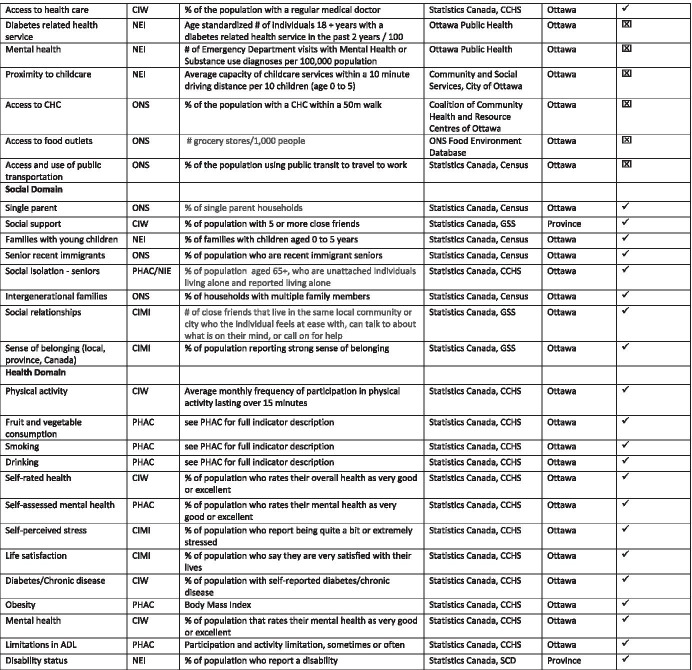
Recommended indicators used by domain, reference, source, and level of disaggregation

*SSHRC* Social Science Humanities Research Council; *ONS* Ottawa Neighbourhood Study; *CIW* Canadian Index of Well-being; *PHAC* Public Health Agency of Canada; *NEI* Ottawa Neighbourhood Equality Index; *CIMI* Canadian Index for Measuring Integration; *LFS* Labour Force Survey; *CCHS* Canadian Community Health Survey; *GSS* General Social Survey; *CSGVP* Canadian Survey of Giving, Volunteering and Participating; *CSD* Canadian Survey on Disability

^1^ONS—Ottawa level data made available through Ottawa Community Data Consortium, Community Data Program of the Canadian Council on Social Development

^2^GSS—Some data may be disaggregated at the level of municipality for immigrants, e.g., # of close friends, sense of belonging, and discrimination

^3^Some data for Ottawa may be further disaggregated by neighborhood (e.g., walk scores)

^4^CCHS—Some data is available for immigrants and racialized groups when cycles are combined (e.g., life satisfaction)

^5^Some data may be disaggregated further by immigrant status (e.g., source country, immigration status) and race (e.g., ethnic group)

Sometimes, additional descriptive data are provided in summary frameworks, such as the statistical definition of quantitative indicators, legal definitions of qualitative indicators, territorial coverage (national provincial levels), temporal coverage (i.e., latest time series’ availability), frequency of data collection, and the periodicity of data availability.

### Identification of Data Gaps

The provision of a working framework and set of indicators represents a first step in addressing how agencies can examine the correlates and consequences of COVID-19 at the local level. However, we also identified several data gaps that need to be addressed to maximize this process. These include gaps related to data collection and those related to data management. Examples of each are identified here.

#### Lack of Disaggregated Population Data

Population data (e.g., Statistics Canada, Canadian Institute for Health Information (CIHI)) were not always disaggregated by race and immigration status. Thus, the heterogeneous nature of immigrants and visible minorities populations is often overlooked (Vang et al., 2017). Disaggregated population data is not easily accessible to non-research agencies. Even in cases where this data is collected, sample sizes do not always permit further disaggregation by specific racialized identities or periods of immigration. One of the only sources of data on immigration category and country of origin is the immigrant landings database (IMDB), and while this data is sometimes linked to other sources of data (e.g., OHIP data in Ontario), linked data sets are not publicly available.

#### Lack of Disaggregated Health Data

Health data is vital to determine if health status/needs, access to health services, and the quality of health services received vary between immigrant and racialized populations. Since provincial and territorial authorities are the primary entities for collecting and reporting on health data in Canada, disaggregated data can vary across Canadian jurisdictions (Blair et al., 2021). Currently, most health facilities do not collect demographic data on racialized or immigrant identities. However, there are some exceptions, e.g., the “We Ask Because We Care” patient demographic tool used by institutions in the former Toronto Central LHIN, Community Health Centres in Ontario. There are also research teams who work with health administrative data linked to the CCHS and/or IMDB who are able to identify health issues specific to immigrant and racialized groups. The collection of disaggregated health data is becoming a major priority in Canada, as evidenced by some of the new initiatives introduced by CIHI. However currently, this data is not easily accessible to community groups.

#### Lack of Community-Level Data

Information on access to services at the community-level data is vital to understand and address local community needs, especially during COVID-19. While some of this data is collected on community institutions, this level of data would ideally include data on access to local resources such as the availability and use of food banks, shelters/hotlines for gender-based violence, recreational programs, seniors’ programs, settlement programs, and educational programs. Many health and community agencies are unable to share their valuable client data because they do not have the capacity to collect this data, or key demographic data is not collected, or they have confidentiality concerns. Data are needed on COVID-19 outcomes disaggregated by workplace (i.e., long-term care facilities) and occupational groups, to identify which sub-populations are experiencing a higher risk of transmission and fatalities.

#### Lack of Individual-Level Data

Information on individual health perceptions and behaviors, especially barriers to help-seeking, can help to develop the types of educational and institutional interventions that may be needed. This type of information would ideally include data on the main modes of COVID-19 transmission in ACB community, COVID-19 testing rates and barriers to getting tested, vaccine hesitancy and uptake, and coping strategies and mental health supports needed.

#### Lack of Local Linked Data

Although there are many sources of data on the social determinants of health at the local level, few of these are linked to public health data, or hospital /health services data, to obtain a fuller picture on the risk and burden of COVID-19 in different population groups and neighborhoods. For example, in Ottawa, Ottawa Neighbourhood Study data includes public health case data; however, associations can only be examined at an ecological level; e.g., neighborhoods with rates of high infection can be determined using its OPH data, and these neighborhoods can be further examined to determine whether they have high proportion of immigrants, racialized groups, seniors, people who are precariously employed, etc.

#### Capacity Challenges

The availability of disaggregated data to community groups does not necessarily ensure that this data will be analyzed and interpreted appropriately. It is important to ensure that community agencies such as OLIP have the resources to hire or train data analysts to work with the data. Often, community groups rely on the “unadjusted” data to make comparisons between populations of interest, but these do not account for other variables such as difference in age, gender, or socioeconomic status, that may explain some of the variations in the outcome of interest between groups. Examining differences between immigrants and non-immigrants fails to consider heterogeneity within the immigrant group. Overlapping and compounding risks related to sex, gender, racialization, income, housing, employment, and other socioeconomic factors are important when examining COVID-19 risk and burden. The ability to integrate intersectional data from various sources, such as self-reported survey data with administrative data, requires adequate expertise, technology, and infrastructure (Statistics Canada, 2019).

## Conclusion and Implications

The current state of race-based data collection about COVID-19 health risks and consequences in federal, provincial, and municipal governments across Canada is a patchwork of measures that lack comparability and comprehensiveness. The decentralized nature of health governance in Canada, combined with the underfunding of public health departments across the country, contributes to the significant gaps in knowledge that we have about racialized health inequities (Van Ingen et al., 2021; Ajadi, 2019; Hyman and Wray, 2014). It is important that available data integrate immigrant and racialized identity variables that capture beyond the binary understanding of immigrant status (i.e., immigrants vs. non-immigrants) and visible minority status (i.e., visible minorities vs. non-visible minorities).

It is timely to note that similar issues have been identified in other jurisdictions. For example, in the USA, the lack of accuracy and incompleteness of race, ethnicity, and language data in health records are long-standing issues and limit progress toward eliminating health inequities (Lurie & Freemont, 2006; Bilheimer & Sisk, 2008). According to Béland (2021), a continuous information system on the organization of prevention and control measures in the settings must already exist and operate before pandemics occur. The importance of this data to understanding COVID-19 cannot be overstated (Wilkins et al., 2021). Immigrant and racialized health inequities will only be eliminated if there is high-quality information by which to track immediate problems and the underlying social determinants of health, to guide the design and implementation of equity-specific medical and public health approaches (Policy Link, 2019). The adoption of standardized tools to collect data on social determinants would enable health systems to integrate health equity into COVID-19 operations to make it a priority, not an isolated stream of work.

The following recommendations are made to policymakers, and community and health stakeholders at all levels, to improve the availability, use, and analysis of disaggregated data to meet the local needs of immigrant and racialized groups who are currently experiencing a higher risk and burden of COVID-19. They reflect the data gaps identified by our team, such as the lack of disaggregated data at various government levels, and the need for capacity building among community and health agencies to do this work. They also reflect the need to include communities in data collection. As captured by the quote, “Nothing about us, without us, is for us” (Charlton, 1998), system transformation must also be driven by the knowledge, voices, and experiences of those who are directly impacted by health inequities.

1. Strategies for community engagement in the collection of disaggregated data
  Continue to raise community awareness about the importance of collecting disaggregated data to document the disproportionate and exacerbated health needs and experiences among immigrant and racialized populations.
  Work with communities and community agencies to address barriers to disaggregated data collection. Research shows that people are willing and able to provide information on their immigrant, racialized status, and other social identities if they understand the purpose of the data collection.
  The design of data collection tools should be informed by the experiences of the community members. Representatives of racialized and immigrant populations should be included in discussions on the development of these tools to ensure that the lived experiences of those disproportionally affected by social determinants of health are fully captured fully.
  Engage with immigrant and racialized communities to ensure that public reporting and data sharing provide appropriate context and interpretation so that data reporting does not stigmatize specific communities but instead is supportive and reflective of their perspectives.
2. Methods for collecting and analyzing data about immigrants and racialized groups
  Consider the adoption of a socio-ecological or other theoretical framework to guide data collection.
  Prioritize data collection among underrepresented groups. This may include the investment in data collection strategies to target communities who are often underrepresented and historically undercounted.
  Work with Statistics Canada and other agencies collecting data to create missing indicators and to develop analytic strategies that better reflect the experiences of immigrant and racialized groups.
  Work with the Canadian Institute for Health Information (CIHI), ICES, and other health agencies to promote the collection of disaggregated data that may be used to examine health access and use, quality of care, and health outcomes among immigrant and racialized groups.
  Work with local community agencies and organizations to promote the collection of disaggregated data that may be used to examine access to services and program outcomes among immigrant and racialized groups. Work with public and community agencies to enhance the collection of disaggregated data. There are many challenges to data collection that compromise data quality and make data collection difficult to implement successfully and sustain over time.
  Recognize the importance of using an intersectional lens in data analysis. Immigrants and racialized populations are not homogenous, and whenever possible, findings should be disaggregated using other social and racialized identities, e.g., country of origin, region, language, and religion.
  Consider data pooling strategies to increase sample sizes to allow a more robust study of the health needs of immigrant and racialized populations during the COVID-19 pandemic. Pooling multiple years of the CCHS relevant to the COVID-19 information would drastically increase the sample size, potentially allowing researchers to produce estimates at the local level.
  Promote and support capacity-building initiatives among community agencies to collect analyze and disseminate data. Sociodemographic data collection and disaggregated data use is complex and requires substantial time, resources, support for implementation, and staff training. Even with consistent, high-quality data, capacity, and resources to analyze the data and apply findings, there are limitations to the analysis that can be done and the information it can provide.
3. Policies to enable and enhance data disaggregation
  Promote the development of policies at all government levels and in all departments to consistently collect disaggregated data as a matter of equity and responsibility.
  Consider promoting the adoption of a disaggregated data collection strategy using human rights principles of participation (i.e., involvement of groups of interest in all aspects of data collection activities), self-identification, transparency, privacy, and accountability (United Nations, 2018). Develop strategies and create opportunities to allow racialized group and immigrant leaders to participate on the decision-making tables, particularly in cities.
  Promote the accessibility and usability of public disaggregated data to community agencies by providing them with capacity-building opportunities and technical support.
  Promote the availability of data on immigrants and racialized groups by supporting data linkage initiatives between government agencies and at the local level.

In conclusion, our working framework, which explicitly recognizes the importance of multi-level data on the social determinants of health to address health inequities, is useful and transferable to other geographic or institutional settings. However, each country or jurisdiction will need to conduct a thorough inventory of their data sources to determine the quality and availability of disaggregated data on SDOH, to identify the most appropriate indicators for their equity-seeking populations, and to take actions (e.g., advocacy, research, policy) to address data gaps.

## Footnotes

1 Health inequities are defined as “differences in health that are not only unnecessary and avoidable but also unfair and unjust” (Whitehead, 1992, p. 430). In this research note, we use the term health inequity, as opposed to health inequality or health disparity, since the latter are limited to epidemiological data (Reutter & Kushner, 2010).
