# Correction to: Strengthening the Collection and Use of Disaggregated Data to Understand and Monitor the Risk and Burden of COVID-19 Among Racialized Populations

**DOI:** 10.1007/s42650-022-00064-4

**Published:** 2022-04-05

**Authors:** Josephine Etowa, Ilene Hyman, Charles Dabone, Ikenna Mbagwu, Bishwajit Ghose, Yujiro Sano, Muna Osman, Hindia Mohamoud

**Affiliations:** 1 Faculty of Health Sciences, University of Ottawa, Ottawa, ON Canada; 2 Dalla Lana School of Public Health, University of Toronto, Toronto, ON Canada; 3 Department of Sociology, Nipissing University, North Bay, ON Canada; 4 Ottawa Local Immigrant Partnership, Ottawa, ON Canada

**Correction to** **:** **Canadian Studies in Population (2021) 48:201–216**

10.1007/s42650-021-00050-2

The original version of this article unfortunately contained an error in Table 1.

The corrected Table 1 is shown below.

**Table 1:** Recommended indicators used by domain, reference, source and level of disaggregation

Domains	Ref	Indicator	Source ^1^	Geography ^2, 3^	Racialized & Immigrant status ^4,5^
Political Domain					
Immigration policies	SSHRC	Existence of policies re: immigration targets, categories, family reunification, settlement services	Gov’t documents	National	x
Poverty reduction polices	SSHRC	Existence of policies e.g., minimum wage, employment insurance, universal daycare, seniors’ benefits, housing, ODSP, etc	Gov’t documents	National	x
Political environment	SSHRC	Global, national, provincial, municipal policies	Gov’t documents	National	x
Economics	SSHRC	Global and local economy, recessions etc	Gov’t documents	National	x
Labour market conditions	SSHRC	Existence of policies re: working conditions, employment in various sectors, unionization	Gov’t documents	National	x
Health policies	SSHRC	Existence of policies e.g., universal coverage, uninsured services	Gov’t documents	National	x
Economic Domain					
Education	ONS	% of pop. without a high school degree	Stats Can, LFS	Ottawa	✓
Education	CIW	% of pop. 25 to 64 yrs with a university degree	Stats Can, LFS	Ottawa	✓
Low income	CIW/NEI	% of pop. that is low income (based on Low Income Cut-Off (LICO))	Stats Can, Canada Income Survey	Ottawa	✓
Employment/Unemployment	PHAC/NEI	% of pop.18–69 that is employed and unemployed	Stats Can, Census	Ottawa	✓
Economic security	CIW	% of labour force that is employed	Stats Can, LFS	Ottawa	✓
Precarious employment	CIW	% of labour force working less than 30 hr per week, not by choice	Stats Can, LFS	Ottawa	✓
Non-precarious employment	CIW	% of labour force with regular, weekday work hours	Stats Can, GSS, Time Use cycles	Province	✓
Low skilled occupation	PHAC	% of pop. 18 to 69 yrs whose current or most relevant recent occupation is classified as NOC skill level / labour force	Stats Can, Census	Ottawa	✓
Occupational mismatch	PHAC	% of pop. 18 to 69 yrs whose highest completed level of education is university and whose current or most relevant recent occupation usually requires high school education or less (NOC levels C & D)	Stats Can, Census	Ottawa	✓
Community Domain					
Social norms and community values	CIW	% of pop. that believes that ‘most or many people can be trusted’	Stats Can, CSGVP	Undetermined	✓
Social engagement	CIW	% of pop. that reports ‘very’ or ‘somewhat’ strong sense of belonging to community	Stats Can, CCHS	Ottawa	✓
Sense of belonging	PHAC	% of pop. that reports a ‘somewhat strong’ or ‘strong’ sense of belonging to their local community	Stats Can, CCHS	Ottawa	✓
Community safety	CIW	% of pop. that feel safe walking alone after dark	Stats Can, GSS	Province	x
Community safety	PHAC	% of pop. 12–89 yrs charged, summed from each of the 5 years	UCR survey	Province	x
Crime against property / individuals	NIE	Crimes against the property/people (total number) per 1,000 people	Ottawa Police Services	Ottawa	x
Crime Severity Index	PHAC	# of police-reported incidents of each of all offence is multiplied by the weight for that offence	UCR survey	Province	x
Food insecurity	CIW	% of households that are food insecure	Stats Can, CCHS	Ottawa	✓
Social exclusion / discrimination	CIW/CIMI	% of pop. that experienced discrimination in past 5 yrs	Stats Can, GSS	Province/Ottawa	✓
Social exclusion / discrimination at work	CIW	% of working pop. 15 + yrs that experienced unfair treatment or discrimination at work	Stats Can, GSS	Province	✓
Housing affordability	PHAC/NEI ONS	% of pop. whose housing is either not affordable, requires major repairs, or size is not sufficient	Stats Can, Census	Ottawa	✓
Subsidized housing	CIMI/ONS	% of renters who live in subsidized housing (i.e., rent applied to income, social housing, public housing, government-assisted housing, or non-profit housing)	Stats Can, Census	Ottawa	✓
Walkability		Ratio of the Sum of Transit/Walkability Score	Walk Score®	Ottawa	x
Commute time	NEI/ONS	% of pop. that spend more than 45 min on their commute	Stats Can, Census	Ottawa	x
Mobility status	NEI	% of pop. that moved in the last 5 years	Stats Can, Census	Ottawa	x
Institutional (Access to Services)					
Access to health care	CIW	% of pop. with a regular medical doctor	Stats Can, CCHS	Ottawa	✓
Diabetes related health service	NEI	Age standardized # of individuals 18 + years with a diabetes related health service in the past 2 years per 100	Ottawa Public Health	Ottawa	x
Mental health	NEI	# of Emergency Department visits with Mental Health or Substance use diagnoses per 100,000 population	Ottawa Public Health	Ottawa	x
Proximity to childcare	NEI	Average capacity of childcare services within a 10 min driving distance per 10 children (0 to 5 yrs)	Community and Social Services, City of Ottawa	Ottawa	x
Access to CHC	ONS	% of pop. with a Community Health Centre within a 50 m walk	Coalition of Community Health and Resource Centres of Ottawa	Ottawa	x
Access to food outlets	ONS	# grocery stores per 1,000 population	ONS Food Environment Database	Ottawa	x
Access and use of public transportation	ONS	% of pop. using public transit to travel to work	Stats Can, Census	Ottawa	x
Social Domain					
Single parent	ONS	% of single parent households	Stats Can, Census	Ottawa	✓
Social support	CIW	% of pop. with 5 or more close friends	Stats Can, GSS	Province	✓
Families with young children	NEI	% of families with children 0 to 5 years	Stats Can, Census	Ottawa	✓
Senior recent immigrants	ONS	% of pop. that are recent immigrant seniors	Stats Can, Census	Ottawa	✓
Social isolation—seniors	PHAC/NIE	% of pop. aged 65 + yrs that are unattached individuals living alone and reported living alone	Stats Can, CCHS	Ottawa	✓
Intergenerational families	ONS	% of households with multiple family members	Stats Can, Census	Ottawa	✓
Social relationships	CIMI	# of close friends that live in the same local community or city who the individual feels at ease with, can talk to about what is on their mind, or call on for help	Stats Can, GSS	Ottawa	✓
Sense of belonging (local, province, Canada)	CIMI	% of pop. that report strong sense of belonging	Stats Can, GSS	Ottawa	✓
Health Domain					
Physical activity	CIW	Average monthly frequency of participation in physical activity lasting over 15 min	Stats Can, CCHS	Ottawa	✓
Fruit & vegetable consumption	PHAC	see PHAC for full indicator description	Stats Can, CCHS	Ottawa	✓
Smoking	PHAC	see PHAC for full indicator description	Stats Can, CCHS	Ottawa	✓
Drinking	PHAC	see PHAC for full indicator description	Stats Can, CCHS	Ottawa	✓
Self-rated health	CIW	% of pop. that rate their overall health as ‘very good’ or ‘excellent’	Stats Can, CCHS	Ottawa	✓
Self-assessed mental health	PHAC/CIW	% of pop. that rate their mental health as ‘very good’ or ‘excellent’	Stats Can, CCHS	Ottawa	✓
Self-perceived stress	CIMI	% of pop. that report being ‘quite a bit’ or’ extremely stressed	Stats Can, CCHS	Ottawa	✓
Life satisfaction	CIMI	% of pop. that report they are very satisfied with their lives	Stats Can, CCHS	Ottawa	✓
Diabetes/Chronic disease	CIW	% of pop. with self-reported diabetes/chronic disease	Stats Can, CCHS	Ottawa	✓
Obesity	PHAC	Body Mass Index	Stats Can, CCHS	Ottawa	✓
Limitations in ADL	PHAC	% of pop. that ‘sometimes’ or ‘often’ report participation and activity limitations	Stats Can, CCHS	Ottawa	✓
Disability status	NEI	% of pop. that report a disability	Stats Can, SCD	Province	✓

*SSHRC* Social Science Humanities Research Council; *ONS* Ottawa Neighbourhood Study; *CIW* Canadian Index of Well-being; *PHAC* Public Health Agency of Canada; *NEI* Ottawa Neighbourhood Equality Index; *CIMI* Canadian Index for Measuring Integration; *LFS* Labour Force Survey; *CCHS* Canadian Community Health Survey; *GSS* General Social Survey; *CSGVP* Canadian Survey of Giving, Volunteering and Participating; *CSD* Canadian Survey on Disability

^1^ONS—Ottawa level data made available through Ottawa Community Data Consortium, Community Data Program of the Canadian Council on Social Development

^2^GSS—Some data may be disaggregated at the level of municipality for immigrants e.g., # of close friends, sense of belonging, discrimination

^3^Some data for Ottawa may be further disaggregated by neighbourhood (e.g. walk scores)

^4^CCHS—Some data is available for immigrants and racialized groups when cycles are combined (e.g. life satisfaction)

^5^Some data may be disaggregated further by immigrant status (e.g. source country, immigration status) and race (e.g., ethnic group)

